# Differential effect of surgical technique on intravesical recurrence after radical nephroureterectomy in patients with upper tract urothelial cancer: a systematic review and Meta-analysis

**DOI:** 10.1007/s00345-024-05185-w

**Published:** 2024-08-20

**Authors:** Ichiro Tsuboi, Akihiro Matsukawa, Mehdi Kardoust Parizi, Jakob Klemm, Robert J Schulz, Anna Cadenar, Stefano Mancon, Sever Chiujdea, Tamás Fazekas, Marcin Miszczyk, Ekaterina Laukhtina, Tatsushi Kawada, Satoshi Katayama, Takehiro Iwata, Kensuke Bekku, Koichiro Wada, Paolo Gontero, Morgan Rouprêt, Jeremy Teoh, Nirmish Singla, Motoo Araki, Shahrokh F. Shariat

**Affiliations:** 1 Department of Urology, Comprehensive Cancer Center Medical University Vienna, Vienna General Hospital, Währinger Gürtel 18-20, Vienna, A-1090 Austria; 2 Department of Urology, Shimane University Faculty of Medicine, Shimane, Japan; 3 Department of Urology, Dentistry and Pharmaceutical Sciences, Okayama University Graduate School of Medicine, Okayama, Japan; 4 Department of Urology, Jikei University School of Medicine, Tokyo, Japan; 5 Department of Urology, Shariati Hospital, Tehran University of Medical Sciences, Tehran, Iran; 6 Department of Urology, University Medical Center Hamburg-Eppendorf, Hamburg, Germany; 7 Unit of Oncologic Minimally Invasive Urology and Andrology, Department of Experimental and Clinical Medicine, Careggi Hospital, University of Florence, Florence, Italy; 8 Department of Biomedical Sciences, Humanitas University, Pieve Emanuele, Italy; 9 Department of Urology, Spitalul Clinic Judetean Murures, University of Medicine, Science, and Technology of Targu Mures, Pharmacy, Mures, Romania; 10 Department of Urology, Semmelweis University, Budapest, Hungary; 11 Collegium Medicum - Faculty of Medicine, WSB University, Dąbrowa Górnicza, Poland; 12 Institute for Urology and Reproductive Health, Sechenov University, Moscow, Russia; 13 Division of Urology, Department of Surgical Sciences, Molinette Hospital, University of Turin, Turin, Italy; 14 Sorbonne University, AP-HP, Pitie-Salpetriere Hospital, GRC 5 Predictive Onco-Uro, Urology, PARIS, F- 75013 France; 15 Department of Surgery, S.H. Ho Urology Centre, The Chinese University of Hong Kong, Hong Kong SAR, China; 16 Department of Urology, James Buchanan Brady Urological Institute, Johns Hopkins University School of Medicine, 600 North Wolfe Street, Park 213, Baltimore, MD 21287 USA; 17 Department of Urology, University of Texas Southwestern Medical Center, Dallas, TX USA; 18 Department of Urology, Weill Cornell Medical College, New York, NY USA; 19 Department of Urology, Second Faculty of Medicine, Charles University, Prague, Czechia Czechia; 20 Division of Urology, Department of Special Surgery, The University of Jordan, Amman, Jordan; 21 Karl Landsteiner Institute of Urology and Andrology, Vienna, Austria; 22 Research Center for Evidence Medicine, Urology Department Tabriz University of Medical Sciences, Tabriz, Iran

**Keywords:** Intravesical recurrence, Radical nephroureterectomy, Upper tract urinary cancer

## Abstract

**Context:**

Radical nephroureterectomy (RNU) with bladder cuff resection is the standard treatment in patients with high-risk upper tract urothelial cancer (UTUC). However, it is unclear which specific surgical technique may lead to improve oncological outcomes in term of intravesical recurrence (IVR) in patients with UTUC.

**Objective:**

To evaluate the efficacy of surgical techniques and approaches of RNU in reducing IVR in UTUC patients.

**Evidence Acquisition:**

Three databases were queried in January 2024 for studies analyzing UTUC patients who underwent RNU. The primary outcome of interest was the rate of IVR among various types of surgical techniques and approaches of RNU.

**Evidence Synthesis:**

Thirty-one studies, comprising 1 randomized controlled trial and 1 prospective study, were included for a systematic review and meta-analysis. The rate of IVR was significantly lower in RNU patients who had an early ligation (EL) of the ureter compared to those who did not (HR: 0.64, 95% CI: 0.44–0.94, *p* = 0.02). Laparoscopic RNU significantly increased the IVR compared to open RNU (HR: 1.28, 95% CI: 1.06–1.54, *p* < 0.001). Intravesical bladder cuff removal significantly reduced the IVR compared to both extravesical and transurethral bladder cuff removal (HR: 0.65, 95% CI: 0.51–0.83, *p* = 0.02 and HR: 1.64, 95% CI: 1.15–2.34, *p* = 0.006, respectively).

**Conclusions:**

EL of the affected upper tract system, ureteral management, open RNU, and intravesical bladder cuff removal seem to yield the lowest IVR rate in patients with UTUC. Well-designed prospective studies are needed to conclusively elucidate the optimal surgical technique in the setting of single post-operative intravesical chemotherapy.

**Supplementary Information:**

The online version contains supplementary material available at 10.1007/s00345-024-05185-w.

## Introduction

Open radical nephroureterectomy (RNU) with complete resection of the ipsilateral bladder cuff is the treatment of choice for high-risk clinically non-metastatic upper tract urothelial cancer (UTUC) [1, 2]. Approximately 30–40% of patients experience intravesical cancer recurrence (IVR) following RNU [3], presumably due to seeding in the majority of cases [4]. Single dose post-operative intravesical chemotherapy is given to lower the risk of IVR based on two prospective trials [5, 6]. Preventing IVR would allow for less intense follow-up, possibly reduce the need for intravesical single dose post-operative chemotherapy and, thereby, lower the cost and burden of care associated with RNU.

To date, several studies [7–12] have reported on the differences in oncological outcomes, including IVR, due to surgical methods or approaches. Grossmann et al. [7] reported there was significant difference in IVR between laparoscopic and open RNU, while Correia et al. [8] showed there was no significant difference between the two groups. As these topics remain controversial, in this systematic review and meta-analysis, we summarize the data on the available surgical methods, and clarify the impact of interventional approach on the risk of IVR of the RNU in patients with UTUC.

## Evidence acquisition

We registered the study with the International Prospective Register of Systematic Reviews (PROSPERO: registration number: CRD42024504011). This systematic review and meta-analysis was conducted in line with the Preferred Reporting Items for Systematic Reviews and Meta-analyses (PRISMA) statement (PRISMA 2020 checklist, Supplementary Table 1).

### Search strategy

On January 2024, the Medline, Scopus, and Web of Science databases were searched to identify studies investigating the impact of surgical technique during RNU on the risk of IVR. The search terms included: “ureteric neoplasms”, “recurrence”, “nephroureterectomy”. The detailed search strategy for each database is shown in the Supplementary Appendix 1. Two investigators independently performed an initial screening based on the titles and abstracts and noted the cause of the exclusion of ineligible reports. Full texts were retrieved and evaluated for eligibility. In addition, searches of reference lists were performed to identify additional studies of interest. In the case of discrepancies, they were solved by consensus among the authors.

### Inclusion and exclusion criteria

We incorporated studies that evaluated IVR following RNU in patients with UTUC. The studies were required to report the surgical technique and the hazard ratio (HR) of IVR following RNU. We excluded studies that lacked original patient data, along with reviews, letters, editorial remarks, responses from authors, case reports, and articles not written in English. When encountering duplicate studies from the same cohorts, we selected either the more recent or the higher-quality publication.

### Data extraction

After duplicate removal, two authors independently screened the titles and abstracts of retrieved records using a standard form. All eligible studies were assessed in full text. The extracted data included: first author, publication year, study region and design, numbers of patients undergoing RNU, and median follow-up time. Additionally, we collected RNU approach, bladder cuff excision method, tumor stage and grade. The endpoint of interest was the rate of IVR reported as hazard ratio (HR) and 95% confidence interval (CI). If the IVR rate was not available in the text, Kaplan-Meier curves were digitized using WebPlotDigitizer software (version 4.6) to extract survival estimates with corresponding 95% CIs [13, 14]. In cases where available graphs did not include 95% CIs, IPDfromKM software was used to reconstruct individual patient data (IPD) based on digitized Kaplan-Meier curves and calculate estimates with corresponding 95% CIs [15].

### Quality assessment & risk of bias

Study quality and risk of bias were evaluated using the Risk-of-Bias (ROB version 2) tool as outlined in the Cochrane Handbook for Systematic Reviews of Interventions [16]. We used the ROBINS-I tool to evaluate bias in non-randomized studies [17]. The RoB2 and ROBINS-I assessment of each study was performed by two authors independently. Finally, we evaluated potential publication bias by using funnel plot and Peters’ linear regression test for funnel plot asymmetry was performed when at least ten studies were included in the meta-analysis.

### Statistical analysis

All statistical analyses were performed using R Version 4.2.2 (R Foundation for Statistical Computing, Vienna, Austria, 2023; meta). To evaluate the effect of the surgical technique on the rate of intravesical recurrence in patients with UTUC who underwent RNU, we generated and analyzed forest plots with HR and 95%CI. For evaluating iRFS, analyses based on contrasts were conducted to estimate the differences in the logarithm of the HR. The standard error was determined using the published HR and CI. Cochrane’s Q test and the I-square test were used to evaluate the heterogeneity with *I*^*2*^ statistics greater than 50% considered significant. When significant heterogeneity was observed, we attempted to investigate the causes of heterogeneity [18]. We performed sensitivity analyses to increase homogeneity and confirm the reliability of our results. P-values at < 0.05 were considered significant.

## Evidence synthesis

### Study selection and characteristics

Our initial search identified 3,039 records. After we removed duplicates, 1,882 records remained for screening of titles and abstracts which led to 1,251 articles being excluded (Supplementary Fig. 1). According to our inclusion criteria, we identified 1 RCT, 1 prospective study, and 29 retrospective studies comprising 20,048 eligible patients for meta-analyses [7, 8, 19–47]. The absolute number of IVR was 5066 patients (25.3%). The median follow-up duration of all patients was 36.1 months ranging from 11.4 to 104.3. The detailed characteristics of the included studies are summarized in Supplementary Table 2.

We found that the surgical relevant steps were generally categorized into three relevant steps for our endpoint of interest: timing of ureter ligation, surgical approach, and technique used for the management of the distal ureter. The timing of ureter ligation was divided into two groups: early ligation (EL) and non-EL (NEL). The definition of EL was generally the ligation of the ureter as soon as possible after starting the surgery or before manipulation of the kidney [20, 25, 31, 38]. The surgical approach was divided into open RNU (ORNU), laparoscopic RNU (LRNU), robot assisted RNU (RANU), and hand-assisted LRNU (H-LRNU). The removal of the distal ureter was divided into an extravesical bladder cuff (EVBC), intravesical bladder cuff (IVBC), transurethral incision bladder cuff (TUBC), and intracorporeal EVBC removal. EVBC was defined as the dissection of the distal ureter and bladder cuff extravesically with lower midline abdominal incision. IVBC was performed generally through a cystotomy procedure with intravesical complete visual excision of the complete intravesical ureter under visual inspection. Intracorporeal EVBC was performed solely with laparoscopic approach, without lower midline abdominal incision.

### Risk of bias assessment

Authors’ judgments about each domain for each included study are graphed in Supplementary Fig. 2 and Supplementary Table 3. Funnel plots and Peter’s Linear Regression analysis are depicted in Supplementary Fig. 3.

### Meta-analysis

**Fig. 1:**
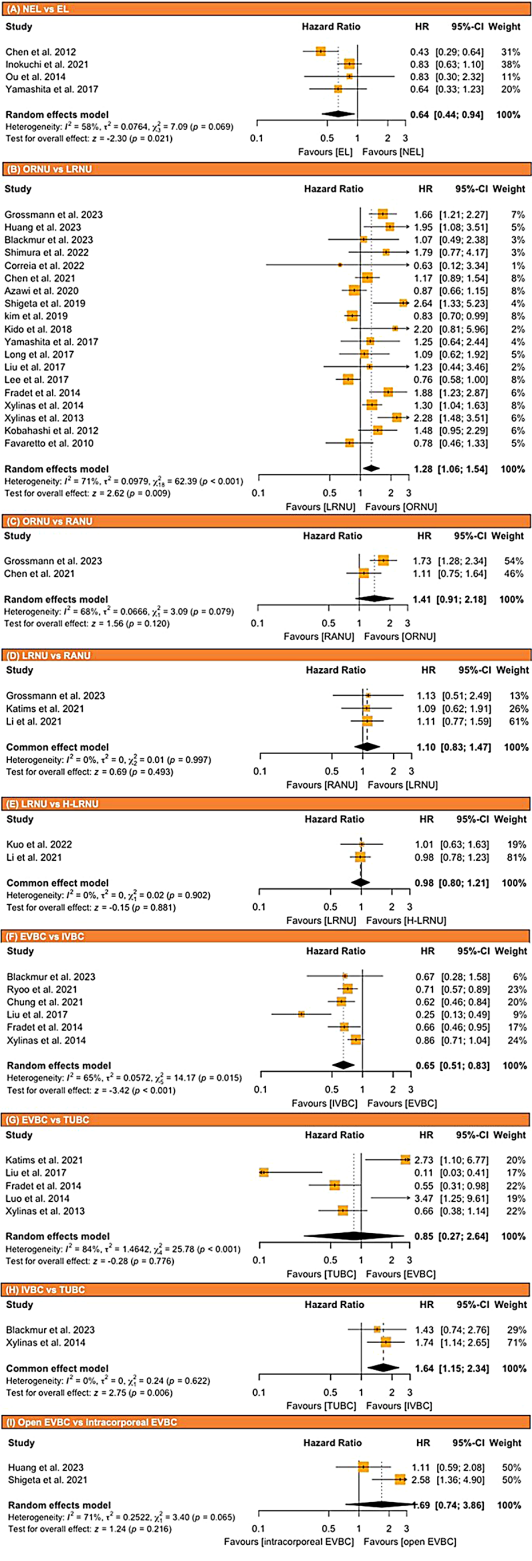
Forest plots showing the effect of surgical technique, approaches, and distal ureter management in preventing intravesical recurrence of UTUC patients who underwent radical nephroureterectomy: **(A)** NEL vs. EL, **(B)** ORNU vs. LRNU, **(C)** ORNU vs. RANU, **(D)** LRNU vs. RANU, **(E)** LRNU vs. H-LRNU, **(F)** EVBC vs. IVBC, **(G)** EVBC vs. TUBC, **(H)** IVBC vs. TUBC, **(I)** Open EVBC vs. intracorporeal EVBC

The results of the meta-analysis are described in Fig. 1.

#### Early ligation (EL) vs no early ligation (NEL) of the ureter

Our analysis comprised four studies: 1 RCT, 1 prospective study, and 2 retrospective studies, with a total of 958 patients [20, 25, 31, 38]. Overall, 390 patients (40.7%) had EL and 568 patients (58.5%) did not. Out of 958 patients, 301 (31.4%) experienced IVR with median follow-up 35 months. The rate of IVR was significantly lower in patients who had an EL compared to those who did not (pooled HR: 0.64, 95% CI: 0.44–0.94, *p* = 0.021; Fig. 1A). The Cochrane’s Q tests and *I*^*2*^ statistic indicated heterogeneity (*p* = 0.07, *I*^*2*^ = 58%). We conducted sensitivity analysis and detected the cause of heterogeneity (Supplementary Fig. 4A).

#### Open RNU (ORNU) vs laparoscopic RNU (LRNU)

Nineteen retrospective studies with a total of 13,243 patients, assessed the impact of LRNU compared to ORNU on IVR. Overall, 2997 of the 13,243 patients (22.6%) experienced IVR. The median follow-up time was 37.7 months. The rate of IVR was significantly higher in patients treated by LRNU compared those treated by ORNU (pooled HR: 1.28, 95% CI: 1.06–1.54, *p* = 0.009; Fig. 1B). The Cochrane’s Q tests and *I*^*2*^ statistic indicated significant heterogeneity (*p* < 0.001, *I*^*2*^ = 71%). Despite conducting a sensitivity analysis, the cause of heterogeneity remained undetected (Supplementary Fig. 4B,4 C,4D). Peter’s Linear Regression analysis did not show the significant difference (*p* = 0.059, Supplementary Fig. 3B).

#### ORNU vs Robot assisted NU (RANU)

Two retrospective studies, comprising 1,144 patients, were analyzed to compare the incidence of IVR between ORNU and RANU [7, 36]. There was no significant difference in the rate of IVR between two groups (pooled HR: 1.41, 95% CIs: 0.91–2.18, *p* = 0.12; Fig. 1C). The Cochrane’s Q tests and *I*^*2*^ statistic indicated heterogeneity (*p* = 0.08, *I*^*2*^ = 68%). Despite conducting a sensitivity analysis, the cause of heterogeneity remained undetected (Supplementary Fig. 4).

#### LRNU vs RANU

An analysis of three studies, comprising 1,588 patients, was conducted to compare the incidence of IVR between LRNU and RANU [7, 39, 40]. LRNU included 799 patients (50.3%) and RANU comprised 789 (49.7%). There was no significant difference in the rate of IVR between the two approaches (pooled HR: 1.10, 95% CI: 0.83–1.47, *p* = 0.5; Fig. 1D). The Cochrane’s Q tests and *I*^*2*^ statistic revealed no significant heterogeneity (*p* = 0.9, *I*^*2*^ = 0%).

#### LRNU vs Hand-assisted LRNU (H-LRNU)

An analysis of two studies [40, 44], comprising 1,521 patients, was conducted to compare the rate of IVR between LRNU and H-LRNU. LRNU included 639 patients (42%), while H-LRNU had 882 (58%). There was no significant difference in the rate of IVR between two groups (pooled HR: 0.98, 95% CI: 0.80–1.21, *p* = 0.9; Fig. 1E). The Cochrane’s Q tests and *I*^*2*^ statistic revealed no significant heterogeneity (*p* = 0.9, *I*^*2*^ = 0%).

#### Extravesical bladder cuff (EVBC) vs intravesical bladder cuff (IVBC)

Six retrospective studies, comprising 5,409 patients, were analyzed to compare the rate of IVR between EVBC and IVBC removal [23, 26, 29, 37, 41, 45]. The rate of IVR was significantly lower in patients who had an IVBC removal compared to those who had an EVBC removal (pooled HR: 0.65, 95% CI: 0.51–0.83, *p* < 0.001; Fig. 1F). The Cochrane’s Q tests and *I*^*2*^ statistic indicated heterogeneity (*p* = 0.015, *I*^*2*^ = 65%). We conducted sensitivity analysis, and the cause of heterogeneity was detected (Supplementary Fig. 4E).

#### EVBC vs TUBC

Five retrospective studies, comprising 1,702 patients, were analyzed to compare the incidence of IVR between EVBC and TUBC removal [23, 24, 26, 29, 39]. There was no significant difference in the rate of IVR between two approaches (pooled HR: 0.85, 95% CI: 0.27–2.64, *p* = 0.8; Fig. 1G). The Cochrane’s Q tests and *I*^*2*^ statistic indicated heterogeneity (*p* < 0.001, *I*^*2*^ = 84%). Despite conducting a sensitivity analysis, the cause of heterogeneity remained undetected (Supplementary Fig. 4F, 4G, 4 H).

#### IVBC vs TUBC

Two retrospective studies, comprising 2,249 patients, were analyzed to compare the incidence of IVR between IVBC and TUBC removal [26, 45]. The rate of IVR was significantly lower in patients who had an IVBC removal compared to those who had a TUBC removal (pooled HR: 1.64, 95% CI: 1.15–2.34, *p* = 0.006; Fig. 1H). The Cochrane’s Q tests and *I*^*2*^ statistic indicated no significant heterogeneity (*p* = 0.6, *I*^*2*^ = 0%).

#### Open EVBC vs Intracorporeal EVBC

Two retrospective studies, comprising 392 patients, were analyzed to compare the incidence of IVR between open and intracorporeal EVBC removal [42, 46]. There was no significant difference in the rate of IVR between two approaches (pooled HR: 1.69, 95% CI: 0.74–3.86, *p* = 0.2; Fig. 1I). The Cochrane’s Q tests and *I*^*2*^ statistic indicated heterogeneity (*p* = 0.06, *I*^*2*^ = 71%).

## Discussion

In this systematic review and meta-analysis, we showed the effect of various surgical techniques during RNU on the rate of IVR in patients treated with RNU for UTUC. Our study revealed several critical findings. First, EL of the ureter during RNU significantly reduces the rate of IVR compared to NEL. Second, LRNU was associated with an increase in the rate of IVR compared to ORNU. Third, IVBC removal was associated with a significant lower rate of IVR in comparison to EVBC or TUBC.

To the best of our knowledge, our meta-analysis is the first to analyze the association between EL and NEL regarding the rate of IVR. Our study indicates that EL of the ureter during RNU with a relative risk reduction of the rate of IVR compared to NEL. Although the pathogenesis of IVR following RNU is still unclear and highly multi factional, one hypothesized mechanism is the downstream seeding of tumor cells from the upper urinary tract into the bladder eventually setting in the bladder mucosa [48]. Therefore, EL of the ureter as soon as possible is plausible to prevent seeding the tumor cells distally. On the other hands, investigator have raised concerns regarding the potential adverse effects of EL of the ureter prior to renovascular ligation on local recurrence and survival rates, due to the risk of direct tumor handling and a significant rise in intrarenal pelvic pressure. Inokuchi et al., for example, revealed that the patients who had an EL had significantly worse in both overall survival (OS) and cancer-specific survival (CSS) compared to those who did not (OS: HR: 1.88, 95%CI 1.24–2.85, *p* = 0.003, CSS: HR: 1.93, 95%CI 1.14–3.25, *p* = 0.014) [38]. Chen et al. reported that EL of the ureter was significantly associated with a lower IVR rate compared to NEL (HR: 0.43, 95%CI 0.29–0.64, *p* = 0.04) [20]. One prospective and two retrospective studies did not a difference, however [25, 31, 38], our analysis supports that EL of the ureter seems to prevent spreading the UTUC to the bladder reducing thereby the IVR rate following RNU, compared to NEL. However, the impact of EL on OS and CSS remains unclear and poorly investigated.

We found that LRNU was associated with a significantly higher risk of IVR compared to ORNU. Seisen et al. [3] reporting that LRNU is indeed associated with an increased rate of IVR compared to ORNU (pooled HR: 1.62, 95%CI: 1.18–2.22, *p* = 0.001). The biology underlying this reality is unclear, but Rouprêt et al. [49] hypothesized that manipulating on UTUC under high intra-abdominal pressure could increases the risk of gravitational seeding with deposition of cancer cells in regions such as the bladder or retroperitoneal space, especially in the context of locally advanced tumors [10, 49]. While RANU should lead to some effect, this has not been shown. This could be due to many other covariates such as EL, differences in the approach to the bladder cuff, and more widely use of intravesical single post-operative chemotherapy in the age of RANU.

We found that IVBC removal leads to lower IVR compared to EVBC and TUBC removal. Complete resection of the distal ureter and the bladder orifice is an integral part of RNU decreasing the risk of IVR [3, 12]. There are several techniques for bladder cuff management, with two previous meta-analyses revealing that EVBC removal is associated with an increased rate of IVR compared to IVBC removal [3, 12]. While an EVBC can obtain a complete removal such as an IVBC, the higher IVR rate is likely due to an incomplete removal in some cases, voluntary or involuntary [12]. TUBC removal has been, similarly, shown to lead to higher IVR rate compared to IVBC removal (pooled HR:1.63. 95%CI: 1.28–2.08, *p* < 0.001). Here again, it is likely that not all the urothelial moiety that is to be removed has eventually been removed. Alternatively or additionally, seeding can have been caused by the TUR process as well [12].

### Limitations

Our study has certain limitations. Firstly, the majority of included studies were retrospective. Additionally, there was significant heterogeneity in some of our analyses. Secondly, our analysis did not perform an analysis regarding the difference in IVR between transperitoneal and retroperitoneal approach. Thirdly, other crucial clinicopathological factors, such as ureteroscopy for definitive diagnosis before RNU, adjuvant intravesical chemotherapy, tumor multiplicity, location, stage, sex, and gender, were not always adjusted for while being considered to be also independent predictors of IVR. Utilizing adjusted HR would have enabled a more accurate interpretation of the results. However, some trials did not conduct or report multivariable analyses. As a result, it was not feasible to conduct an adjusted meta-analysis.

## Conclusions

We found that EL of the ureter significantly reduced the risk of IVR in patients with UTUC following RNU. LRNU lead to significantly increased of the risk of IVR compared to ORNU. IVBC removal significantly decreased the rate of IVR compared to both EVBC or TUBC removal. Our analyses mostly included retrospective studies, further studies are needed to clarify the benefit of surgical techniques and approaches to reduce the IVR rate in patients with UTUC following RNU in the age of simple post-operative intravesical chemotherapy. These three concrete and valid steps can serve as a basis for quality indicators in the management of high-risk UTUC.

Figure 1. Forest plots showing the effect of surgical technique, approaches, and distal ureter management in preventing intravesical recurrence of UTUC patients who underwent radical nephroureterectomy: (A) NEL vs. EL, (B) ORNU vs. LRNU, (C) ORNU vs. RANU, (D) LRNU vs. RANU, (E)LRNU vs. H-LRNU, (F) EVBC vs. IVBC, (G) EVBC vs. TUBC, (H) IVBC vs. TUBC, (I) Open EVBC vs. intracorporeal EVBC.

EL: early ligation, EVBC: extravesical bladder cuff, IVBC: intravesical bladder cuff, H-LRNU = hand-assisted laparoscopic radical nephroureterectomy, LRNU: laparoscopic radical nephroureterectomy, ORNU: open radical nephroureterectomy, RANU: robot assisted radical nephroureterectomy, RNU: radical nephroureterectomy, TUBC: transurethral incision of the bladder cuff, UTUC: upper tract urothelial cancer.

## Electronic supplementary material

Below is the link to the electronic supplementary material.

Supplementary Material 1
