# Supplementary material for: Differential effect of surgical technique on intravesical recurrence after radical nephroureterectomy in patients with upper tract urothelial cancer: a systematic review and Meta-analysis
Source: World J Urol. 2024 Aug 20;42(1):488. doi: 10.1007/s00345-024-05185-w (PMC11335797; doi:10.1007/s00345-024-05185-w)
Supplement: Supplementary file 1 — Supplementary Material 1 [file 345_2024_5185_MOESM1_ESM.docx]

**Supplementary Information**

**Supplementary Appendix 1**. Search strategy for meta-analysis

**Supplementary Table 1.** PRISMA checklist 2020

**Supplementary Table 2.** Characteristics of included studies

**Supplementary Table 3.** ROBINS-I

**Supplementary Figure 1.** PRISMA flowchart

**Supplementary Figure 2.** Risk of bias assessment of the included RCTs

**Supplementary Figure 3.** Funnel plots and Peter’s Linear Regression analysis

**Supplementary Figure 4.** Sensitivity analysis

**Supplementary Appendix 2**. AMSTAR-2 checklist

**Abbreviations:**

CSS = cancer-specific survival

EVBC = extravesical bladder cuff

EL = early ligation

IVBC = intravesical bladder cuff

IVR = intravesical recurrence

H-LRNU = hand-assisted laparoscopic radical nephroureterectomy

LRNU =laparoscopic radical nephroureterectomy

NEL = non early ligation

ORNU = open radical nephroureterectomy

OS = overall survival

RANU = robot assisted radical nephroureterectomy

RFS = recurrence-free survival

RNU = radical nephroureterectomy

PRISMA = preferred reporting items for systematic reviews and meta-analyses

ROB2 = risk of bias version 2

ROBINS-I = risk of bias in non-randomized studies of interventions

TUBC = transurethral incision of the bladder cuff

UTUC = upper tract urothelial cancer

**Supplementary Appendix 1**. Search strategy for systematic review and meta-analysis

Electronic searches were performed in the following databases to identify eligible studies.

1. PubMed (n = 857)
2. SCOPUS (n = 1457)
3. Web of science (n = 725)

There were no language or publication period limitations.

PubMed

1. Search: recurrence [Title/Abstract]
2. Search: nephroureterectomy [Title/Abstract]
3. Search: ureteric neoplasms [MeSHTerms]
4. Search: “transitional cell carcinoma” [Title/Abstract]
5. #3 OR #4
6. #1 AND #2 AND #5

SCOPUS

 TITLE-ABS-KEY ( "recurrence" ) AND TITLE-ABS-KEY ( "nephroureterectomy" ) AND ( TITLE-ABS-KEY ( "ureteric neoplasms" ) OR TITLE-ABS-KEY ( "transitional cell carcinoma" ) )

Web of science

TS=("recurrence") AND TS=("nephroureterectomy") AND (TS=("ureteric neoplasms") OR TS=("transitional cell carcinoma"))

**Supplementary Table 1.** PRISMA checklist 2020

| **Section and Topic** | **Item #** | **Checklist item** | **Location where item is reported** |
| --- | --- | --- | --- |
| **TITLE** | | |  |
| Title | 1 | Identify the report as a systematic review. | 1 |
| **ABSTRACT** | | |  |
| Abstract | 2 | See the PRISMA 2020 for Abstracts checklist. | 4-5 |
| **INTRODUCTION** | | |  |
| Rationale | 3 | Describe the rationale for the review in the context of existing knowledge. | 6 |
| Objectives | 4 | Provide an explicit statement of the objective(s) or question(s) the review addresses. | 6 |
| **METHODS** | | |  |
| Eligibility criteria | 5 | Specify the inclusion and exclusion criteria for the review and how studies were grouped for the syntheses. | 6-9 |
| Information sources | 6 | Specify all databases, registers, websites, organisations, reference lists and other sources searched or consulted to identify studies. Specify the date when each source was last searched or consulted. | 6-9 |
| Search strategy | 7 | Present the full search strategies for all databases, registers and websites, including any filters and limits used. | 6-9 |
| Selection process | 8 | Specify the methods used to decide whether a study met the inclusion criteria of the review, including how many reviewers screened each record and each report retrieved, whether they worked independently, and if applicable, details of automation tools used in the process. | 6-9 |
| Data collection process | 9 | Specify the methods used to collect data from reports, including how many reviewers collected data from each report, whether they worked independently, any processes for obtaining or confirming data from study investigators, and if applicable, details of automation tools used in the process. | 6-9 |
| Data items | 10a | List and define all outcomes for which data were sought. Specify whether all results that were compatible with each outcome domain in each study were sought (e.g. for all measures, time points, analyses), and if not, the methods used to decide which results to collect. | 6-9 |
|  | 10b | List and define all other variables for which data were sought (e.g. participant and intervention characteristics, funding sources). Describe any assumptions made about any missing or unclear information. | 6-9 |
| Study risk of bias assessment | 11 | Specify the methods used to assess risk of bias in the included studies, including details of the tool(s) used, how many reviewers assessed each study and whether they worked independently, and if applicable, details of automation tools used in the process. | 9 |
| Effect measures | 12 | Specify for each outcome the effect measure(s) (e.g. risk ratio, mean difference) used in the synthesis or presentation of results. | 6-9 |
| Synthesis methods | 13a | Describe the processes used to decide which studies were eligible for each synthesis (e.g. tabulating the study intervention characteristics and comparing against the planned groups for each synthesis (item #5)). | 6-9 |
|  | 13b | Describe any methods required to prepare the data for presentation or synthesis, such as handling of missing summary statistics, or data conversions. | 6-9 |
|  | 13c | Describe any methods used to tabulate or visually display results of individual studies and syntheses. | 6-9 |
|  | 13d | Describe any methods used to synthesize results and provide a rationale for the choice(s). If meta-analysis was performed, describe the model(s), method(s) to identify the presence and extent of statistical heterogeneity, and software package(s) used. | 6-9 |
|  | 13e | Describe any methods used to explore possible causes of heterogeneity among study results (e.g. subgroup analysis, meta-regression). | 6-9 |
|  | 13f | Describe any sensitivity analyses conducted to assess robustness of the synthesized results. | 6-9 |
| Reporting bias assessment | 14 | Describe any methods used to assess risk of bias due to missing results in a synthesis (arising from reporting biases). | 6-9 |
| Certainty assessment | 15 | Describe any methods used to assess certainty (or confidence) in the body of evidence for an outcome. | 6-9 |
| **RESULTS** | | |  |
| Study selection | 16a | Describe the results of the search and selection process, from the number of records identified in the search to the number of studies included in the review, ideally using a flow diagram. | 9-10 |
|  | 16b | Cite studies that might appear to meet the inclusion criteria, but which were excluded, and explain why they were excluded. | 9-10 |
| Study characteristics | 17 | Cite each included study and present its characteristics. | 9-10, Table 1 |
| Risk of bias in studies | 18 | Present assessments of risk of bias for each included study. | 10-11, Supplementary Figure1 and Supplementary Table2 |
| Results of individual studies | 19 | For all outcomes, present, for each study: (a) summary statistics for each group (where appropriate) and (b) an effect estimate and its precision (e.g. confidence/credible interval), ideally using structured tables or plots. | 11-12 |
| Results of syntheses | 20a | For each synthesis, briefly summarise the characteristics and risk of bias among contributing studies. | 11-12 |
|  | 20b | Present results of all statistical syntheses conducted. If meta-analysis was done, present for each the summary estimate and its precision (e.g. confidence/credible interval) and measures of statistical heterogeneity. If comparing groups, describe the direction of the effect. | 11-12 |
|  | 20c | Present results of all investigations of possible causes of heterogeneity among study results. | Supplementary figure 3 |
|  | 20d | Present results of all sensitivity analyses conducted to assess the robustness of the synthesized results. | 11-12 |
| Reporting biases | 21 | Present assessments of risk of bias due to missing results (arising from reporting biases) for each synthesis assessed. | Supplementary Figure 1 and Supplementary Table 2 |
| Certainty of evidence | 22 | Present assessments of certainty (or confidence) in the body of evidence for each outcome assessed. | 11-12 |
| **DISCUSSION** | | |  |
| Discussion | 23a | Provide a general interpretation of the results in the context of other evidence. | 12-13 |
|  | 23b | Discuss any limitations of the evidence included in the review. | 12-13 |
|  | 23c | Discuss any limitations of the review processes used. | 12-13 |
|  | 23d | Discuss implications of the results for practice, policy, and future research. | 12-13 |
| **OTHER INFORMATION** | | |  |
| Registration and protocol | 24a | Provide registration information for the review, including register name and registration number, or state that the review was not registered. | NA |
|  | 24b | Indicate where the review protocol can be accessed, or state that a protocol was not prepared. | NA |
|  | 24c | Describe and explain any amendments to information provided at registration or in the protocol. | NA |
| Support | 25 | Describe sources of financial or non-financial support for the review, and the role of the funders or sponsors in the review. | Authorship form |
| Competing interests | 26 | Declare any competing interests of review authors. | Authorship form |
| Availability of data, code and other materials | 27 | Report which of the following are publicly available and where they can be found: template data collection forms; data extracted from included studies; data used for all analyses; analytic code; any other materials used in the review. | NA |

**Supplementary Table 2.** Characteristics of included studies

| **No** | **Author** | **year** | **Study design** | **Period** | **No. of patients** | **Median follow-up, mo** | | | **No. of IVR (%)** | | **Median time to IVR, mo** | | |
| --- | --- | --- | --- | --- | --- | --- | --- | --- | --- | --- | --- | --- | --- |
| **Pooled results** | | | | | **20048** | **36.1** | **(11.4** | **-104.3)** | **5066** | **(25.3)** | **14** | **(4.3** | **-30.5)** |
| 1 | Grossmann et al. | 2023 | PSM | 1990-2020 | 756 | 32 | (15 | -61) | 292 | (38.6) | NA | | |
| 2 | Blackmur et al. | 2023 | retrospective | 2012-2019 | 402 | 29 | (16 | -54) | 114 | (28.4) | NA | | |
| 3 | Huang et al. | 2023 | retrospective | 2010-2020 | 452 | 29.5 | (15.1 | -52) | 134 | (29.6) | 7.6 | (3.6 | -13.1) |
| 4 | Shimura et al. | 2022 | retrospective | 1990-2015 | 403 | 34.2 | (12.9 | -75.5) | 164 | (40.7) | 8.4 | (4.8 | -18.3) |
| 5 | Kuo et al. | 2022 | retrospective | 1988-2020 | 322 | NA | | | 230 | (71.4) | NA | | |
| 6 | Haung et al. | 2022 | retrospective | 2005-2020 | 1239 | NA | | | 342 | (27.6) | NA | | |
| 7 | Correia et al. | 2022 | retrospective | 2006-2020 | 48 | 21 | (6 | -54) | 5 | (10.4) | NA | | |
| 8 | Katims et al. | 2021 | retrospective | 2015-2019 | 485 | 20.5 |  |  | 110 | (22.7) | 15.2 |  |  |
| 9 | Inokuchi et al. | 2021 | retrospective | 1995-2009 | 664 | 46.8 | (25.2 | -75.6) | 218 | (32.8) | NA | | |
| 10 | Shigeta et al. | 2021 | PSM | 2006-2018 | 136 | 26 | (3.3 | -135.1) | 51 | (37.5) | NA | | |
| 11 | Ryoo et al. | 2021 | retrospective | 1992-2018 | 865 | 37.7 | (15.9 | -74.4) | 320 | (37) | 17.9 | (7.1 | -47.9) |
| 12 | Chung et al. | 2021 | retrospective | 1994-2018 | 1095 | NA | | | 462 | (42.2) | 13.8 | (3.9 | -14.1) |
| 13 | Chen et al. | 2021 | retrospective | 1998-2021 | 1808 | 41.8 |  |  | 482 | (26.7) | NA | | |
| 14 | Li et al. | 2021 | retrospective | 2001-2021 | 741 | NA | | | NA | | NA | | |
| 15 | Azawi et al. | 2020 | retrospective | 2004-2017 | 1384 | 54 | (28 | -82) | 292 | (21.1) | 9 | (5 | -19) |
| 16 | Shigeta et al. | 2019 | PSM | 1990-2015 | 144 | 65.4 | (3.8 | -316.9) | 44 | (30.7) | NA | | |
| 17 | kim et al. | 2019 | PSM | 2000-2012 | 1276 | 40.45 | (0.1 | -184.4) | NA | | NA | | |
| 18 | Kido et al. | 2018 | retrospective | 1995-2017 | 426 | NA | | | NA | | NA | | |
| 19 | Long et al. | 2017 | retrospective | 2004-2012 | 685 | 47 | (36 | -62) | 159 | (23.2) | 11 | (3 | -35) |
| 20 | Liu et al. | 2017 | retrospective | 2000-2013 | 265 | 60 |  |  | 46 | (17.4) | NA | | |
| 21 | Lee et al. | 2017 | retrospective | 2000-2015 | 760 | 45 | (3 | -76) | 231 | (30.4) | 10 | (3 | -37) |
| 22 | Yamashita et al. | 2017 | Prospective | 2012-2013 | 148 | 24 |  |  | 44 | (29.7) | NA | | |
| 23 | Yamashita et al. | 2016 | retrospective | 2000-2011 | 534 | 47 | (1 | -178) | 205 | (38.4) | NA | | |
| 24 | Fradet et al. | 2014 | retrospective | 1990-2010 | 743 | 40.4 | (7.7 | -56.8) | 167 | (22.5) | 17.2 | (5.4 | -45.4) |
| 25 | Xylinas et al. | 2014 | retrospective | 1987-2007 | 2681 | 57.5 | (1 | -271) | 577 | (21.5) | 38.3 |  |  |
| 26 | Ou et al. | 2014 | retrospective | 2003-2011 | 61 | 39.7 | (12 | -96) | 18 | (29.5) | NA | | |
| 27 | Luo et al. | 2014 | retrospective | 2005-2010 | 396 | 40.65 |  |  | NA | | NA | | |
| 28 | Xylinas et al. | 2013 | retrospective | 1995-2009 | 482 | 39.5 | (25 | -60) | 169 | (35.1) | NA | | |
| 29 | Chen et al. | 2012 | RCT | 2007-2009 | 85 | 28 | (5 | -60) | 21 | (24.7) | 13 | (5 | -24) |
| 30 | Kobahashi et al. | 2012 | retrospective | 2005-2009 | 288 | 20.2 | (3 | -61.6) | 103 | (35.8) | 6.9 | (3 | -51.3) |
| 31 | Favaretto et al. | 2010 | retrospective | 1995-2008 | 274 | 23 |  |  | 66 | (24.2) | NA | | |

**Supplementary Table 3.** Risk of bias assessment for individual studies using the Risk of Bias in non-randomized controlled studies-of interventions tool (ROBINS-I)

| Author | Confounding | Classification of interventions | Deviations from intender intervention | Missing data | Measurement of outcomes | Selection of the reported result | Overall |
| --- | --- | --- | --- | --- | --- | --- | --- |
| Grossmann et al. | Low | Low | Low | Moderate | Moderate | Low | Moderate |
| Blackmur et al. | Moderate | Moderate | Moderate | Moderate | Moderate | Moderate | Moderate |
| Huang et al. | Moderate | Moderate | Moderate | Moderate | Moderate | Moderate | Moderate |
| Shimura et al. | Moderate | Low | Low | Moderate | Moderate | Low | Moderate |
| Kuo et al. | Moderate | Moderate | Moderate | Moderate | Moderate | Moderate | Moderate |
| Haung et al. | Moderate | Moderate | Moderate | Low | Moderate | Moderate | Moderate |
| Correia et al. | Moderate | Moderate | Moderate | Low | Low | Moderate | Moderate |
| Katims et al. | Moderate | Moderate | Moderate | Low | Moderate | Moderate | Moderate |
| Inokuchi et al. | Moderate | Moderate | Moderate | Moderate | Moderate | Moderate | Moderate |
| Shigeta et al. | Low | Moderate | Moderate | Moderate | Moderate | Moderate | Moderate |
| Ryoo et al. | Moderate | Moderate | Moderate | Low | Moderate | Moderate | Moderate |
| Chung et al. | Moderate | Moderate | Moderate | Low | Moderate | Moderate | Moderate |
| Chen et al. | Moderate | Moderate | Moderate | Moderate | Moderate | Moderate | Moderate |
| Li et al. | Moderate | Moderate | Moderate | Moderate | Moderate | Moderate | Moderate |
| Azawi et al. | Moderate | Moderate | Moderate | Low | Moderate | Moderate | Moderate |
| Shigeta et al. | Low | Moderate | Moderate | Low | Low | Moderate | Moderate |
| kim et al. | Low | Moderate | Moderate | Low | Low | Moderate | Moderate |
| Kido et al. | Moderate | Moderate | Moderate | Moderate | Moderate | Moderate | Moderate |
| Long et al. | Moderate | Moderate | Moderate | Low | Moderate | Moderate | Moderate |
| Liu et al. | Moderate | Moderate | Moderate | Moderate | Moderate | Moderate | Moderate |
| Lee et al. | Moderate | Moderate | Moderate | Low | Moderate | Moderate | Moderate |
| Yamashita et al. | Low | Moderate | Moderate | Moderate | Low | Low | Moderate |
| Yamashita et al. | Moderate | Moderate | Moderate | Moderate | Moderate | Moderate | Moderate |
| Fradet et al. | Moderate | Moderate | Moderate | Low | Moderate | Moderate | Moderate |
| Xylinas et al. | Moderate | Moderate | Moderate | Low | Moderate | Moderate | Moderate |
| Ou et al. | Moderate | Moderate | Moderate | Moderate | Moderate | Moderate | Moderate |
| Luo et al. | Moderate | Moderate | Moderate | Moderate | Moderate | Moderate | Moderate |
| Xylinas et al. | Moderate | Moderate | Moderate | Moderate | Moderate | Moderate | Moderate |
| Kobahashi et al. | Moderate | Moderate | Moderate | Low | Moderate | Moderate | Moderate |
| Favaretto et al. | Moderate | Moderate | Moderate | Moderate | Moderate | Moderate | Moderate |

**Supplementary Figure 1.** The Preferred Reporting Items for Systematic Reviews and Meta-analyses (PRISMA) flow chart, detailing the article selection process

**Supplementary Figure 2.** Risk of bias assessment of the included RCTs


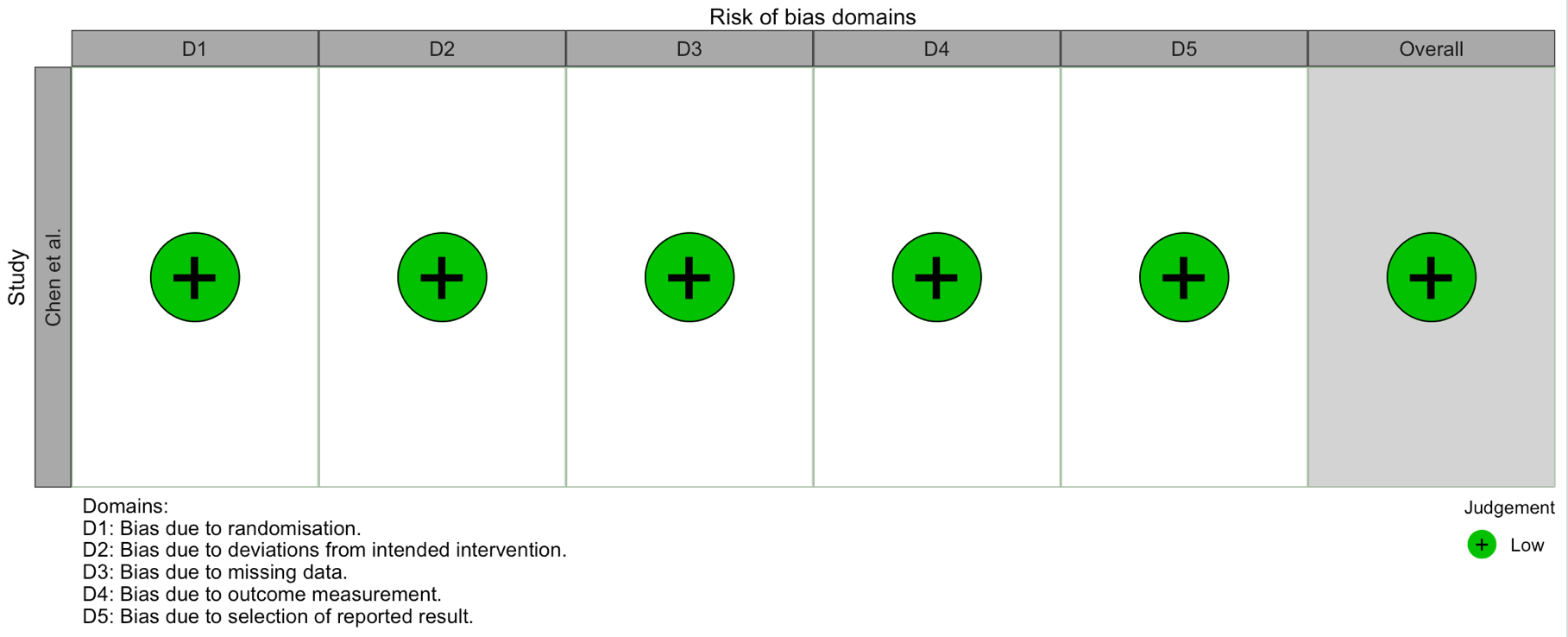


**Supplementary Figure 3.** Funnel Plot

1. EL vs NEL

1. ORNU vs LRNU

1. ORNU vs RANU

1. LRNU vs RANU

1. LRNU vs H-LRNU

1. EVBC vs IVBC

1. EVBC vs TUBC

1. IVBC vs TUBC

1. Open EVBC cs Intracorporeal EVBC

Supplementary Figure 4. Sensitivity analysis

1. Forest plots showing the impact of EL of ureter during nephroureterectomy about IVR without Chen et al.

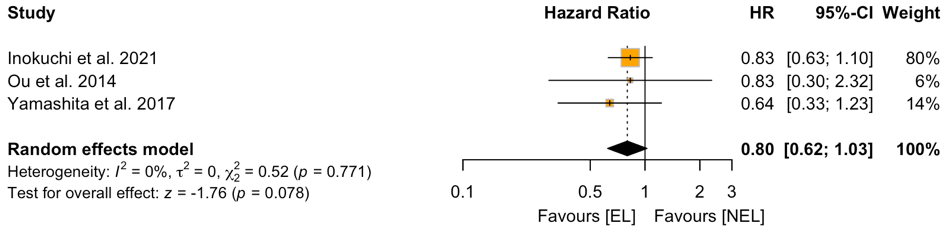

2. Forest plots showing the impact of surgical approach between ORNU and LRNU about IVR without Correia et al.

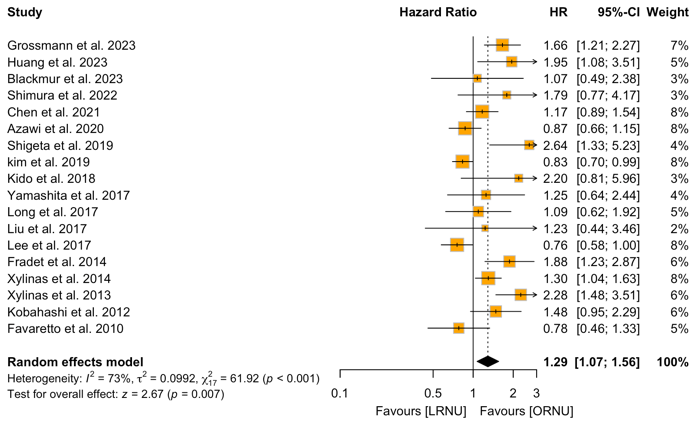

3. Forest plots showing the impact of surgical approach between ORNU and LRNU about IVR without Favaretto et al.

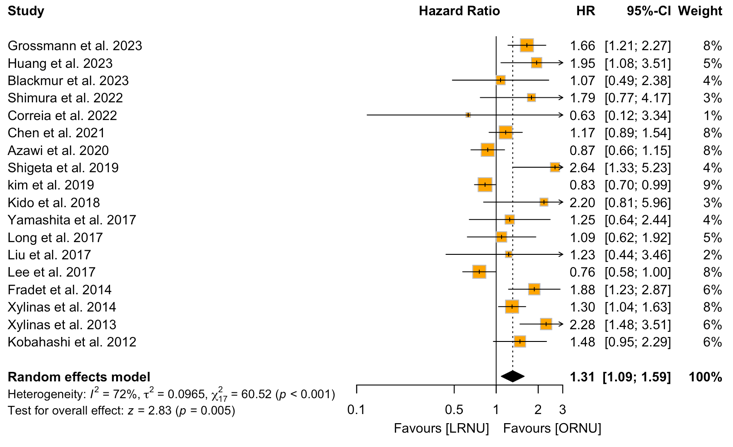

4. Forest plots showing the impact of surgical approach between ORNU and LRNU about IVR without Lee et al.


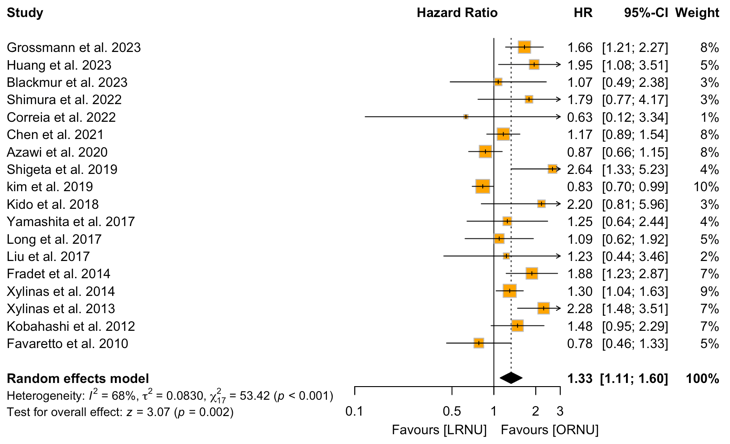


1. Forest plots showing the impact of management of distal ureter between EVBC and IVBC about IVR without Liu et al.


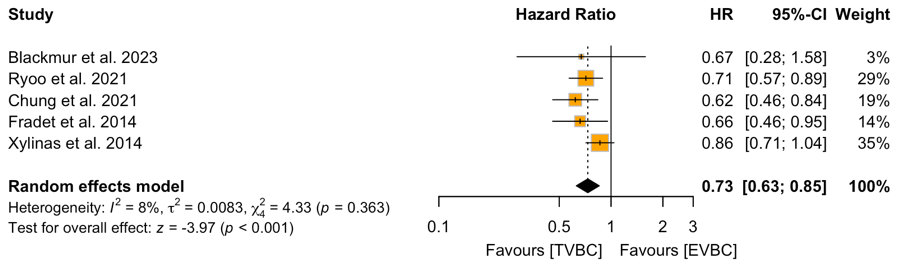


1. Forest plots showing the impact of management of distal ureter between EVBC and TUBC about IVR without katims et al.


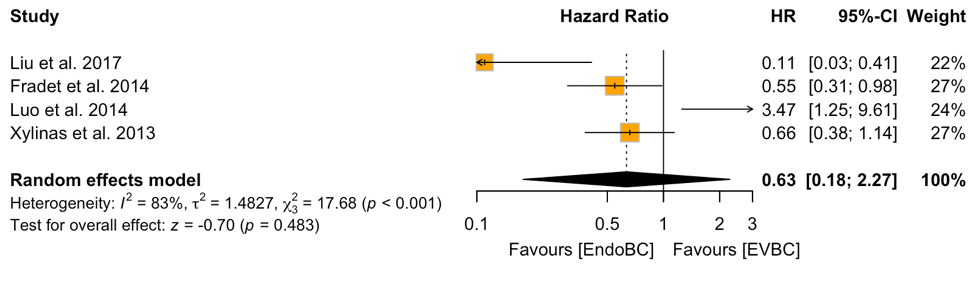


1. Forest plots showing the impact of management of distal ureter between EVBC and TUBC about IVR without Luo et al.


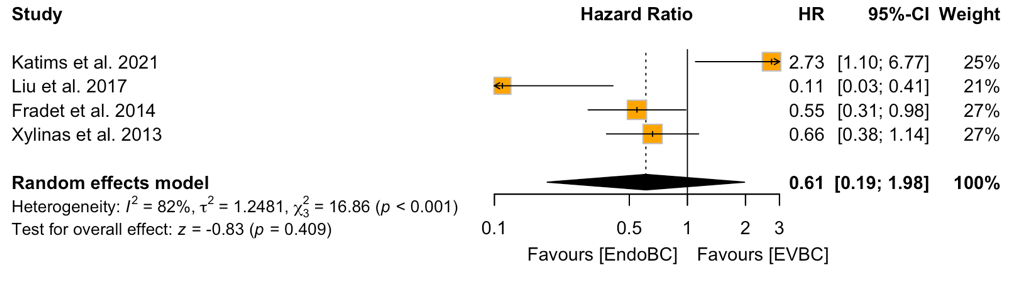


1. Forest plots showing the impact of management of distal ureter between EVBC and TUBC about IVR without Liu et al.


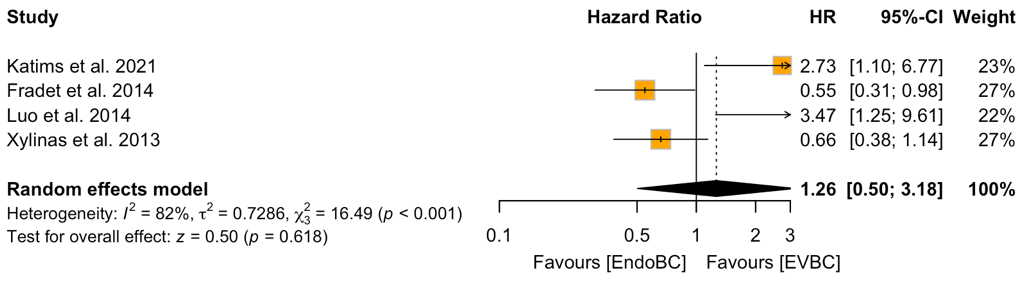


**Supplementary Appendix 2**. AMSTAR-2 checklist


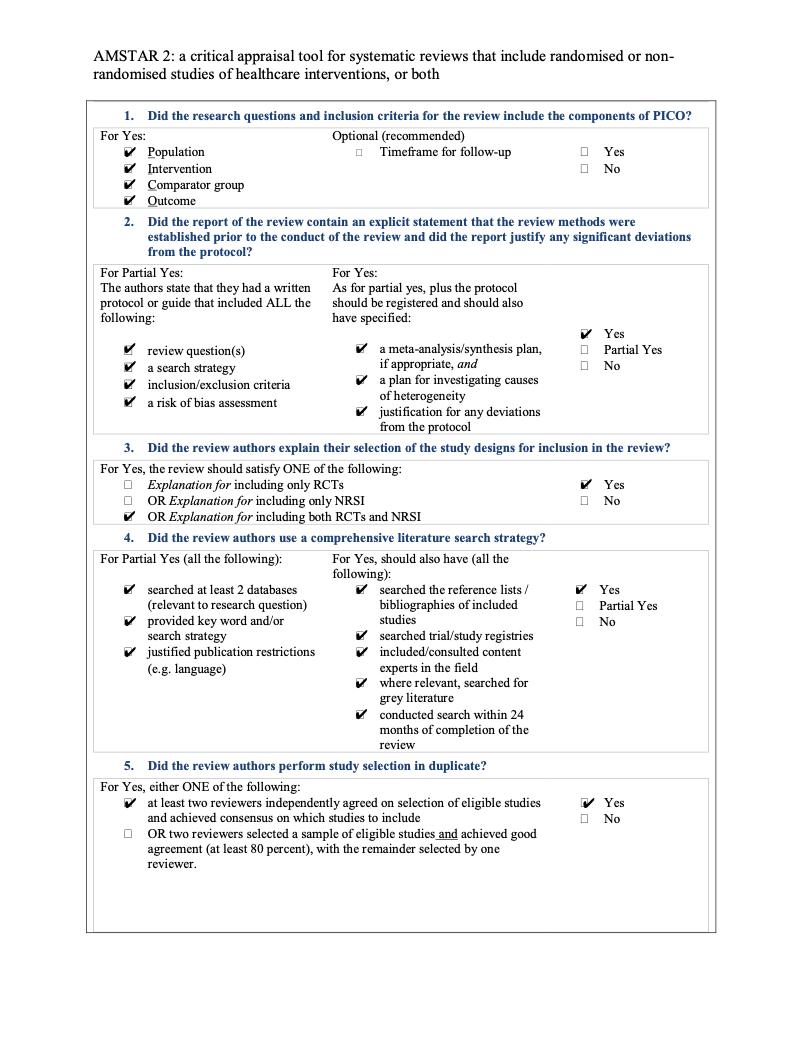


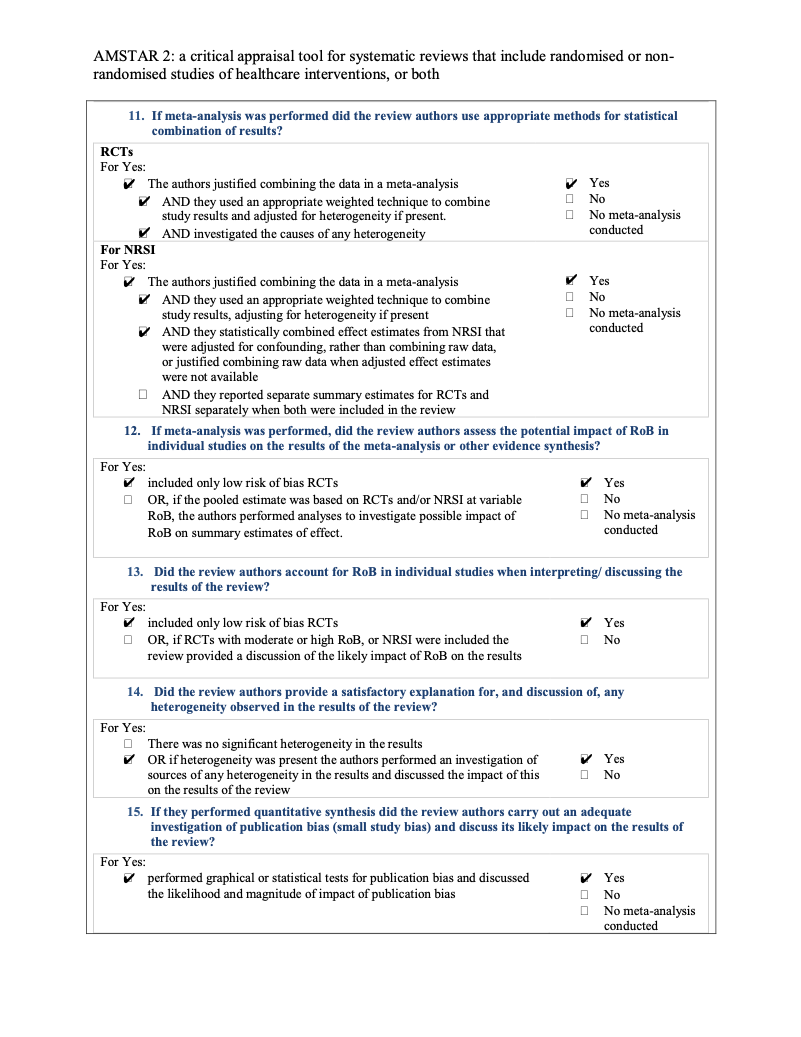


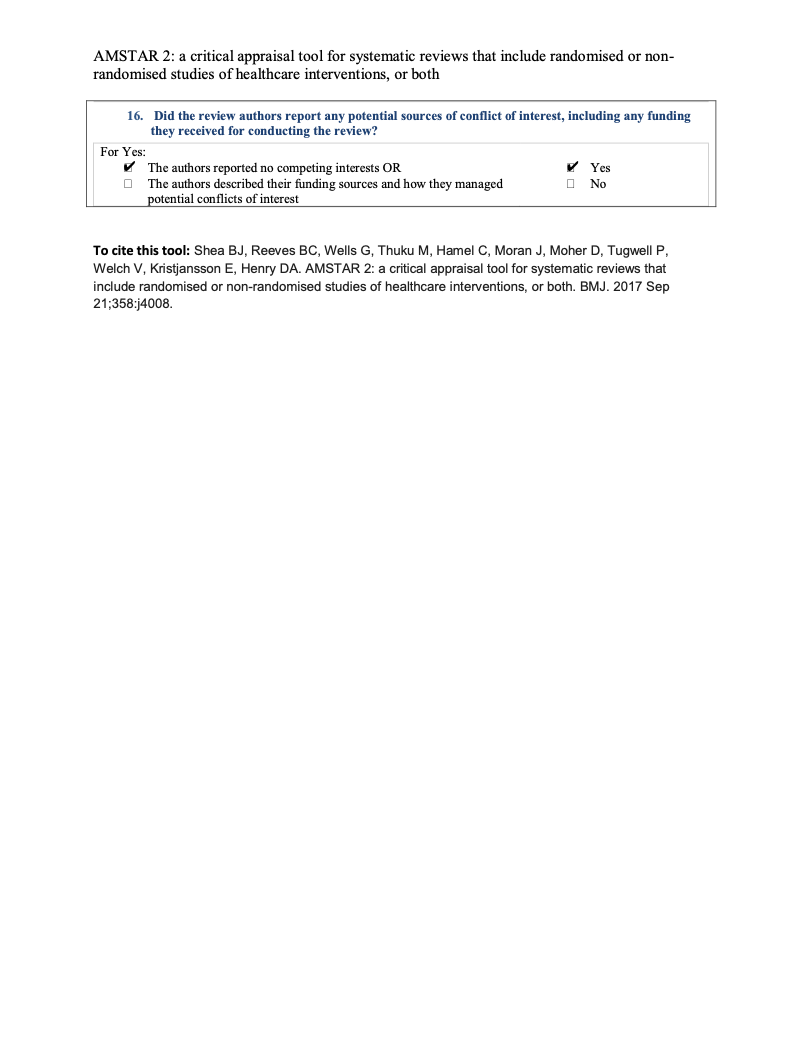


References
